# Delayed Paraplegia after Complex Repair of Thoracic Aortic Dissection

**DOI:** 10.1055/a-2524-4880

**Published:** 2025-02-28

**Authors:** Daniel Nguyen, Scott S. Berman, Luis R. Leon

**Affiliations:** 1 The University of Arizona Section of Vascular Surgery, Tucson, Arizona; 2 Southern Arizona Vascular Institute, Tucson, Arizona

**Keywords:** spinal cord ischemia, delayed paraplegia

## Abstract

Spinal cord ischemia (SCI) is a well-known complication of both open and endovascular repair of the thoracoabdominal aorta. Perioperative maneuvers to increase spinal cord perfusion, including permissive hypertension and lumbar drain placement to control spinal pressure, are commonly used to reduce the risk of SCI. Additional perioperative measures to reduce the susceptibility of the spinal cord to ischemic insult include hypothermia, steroids, and naloxone infusion. Most cases manifest immediately or within days of surgery and improve with the aforementioned maneuvers. We describe a rare occurrence of delayed SCI 20 months after thoracic endovascular aortic aneurysm repair.

Spinal cord ischemia (SCI) is a rare, but serious complication of thoracic endovascular aortic aneurysm repair (TEVAR) associated with debilitating paraplegia. Perfusion of the spinal cord is normally maintained by a dynamic network of single segmental arteries and collateral circulation comprised of the subclavian, intercostal, hypogastric, and Adamkiewicz arteries. 1 2 In TEVAR, the obliteration of segmental branches can normally be compensated for by adjacent uncovered collateral vessels. 3 Nevertheless, any disturbance to this tenuous blood supply may induce profound enough hypoperfusion to precipitate SCI. 4

TEVAR theoretically avoids several critical hemodynamic changes implicated in SCI that occur during open repair, including aortic cross-clamping and reperfusion injury. 5 The likelihood of developing post-TEVAR SCI is lower than open repair but still occurs at an incidence of 2 to 15%. 6 Furthermore, the risk of SCI is considered highest during the 30-day perioperative period. 7 The extent of neurologic deficits associated with SCI ranges from mild paraparesis with potential for recovery, all the way up to complete paralysis from irreversible ischemia.

Compared with immediate SCI, which is observed shortly after emergence from anesthesia, delayed-onset SCI is an underreported phenomenon that follows a period of normal neurologic function and is considered secondary to a postoperative event. The timing of delayed-onset SCI remains variable, with the literature describing occurrences from several weeks up to 19 months postoperatively. 2 3 8 9 10 We present a rare case of delayed SCI occurring 20 months after TEVAR.

## Case Review

**Fig. 1:**
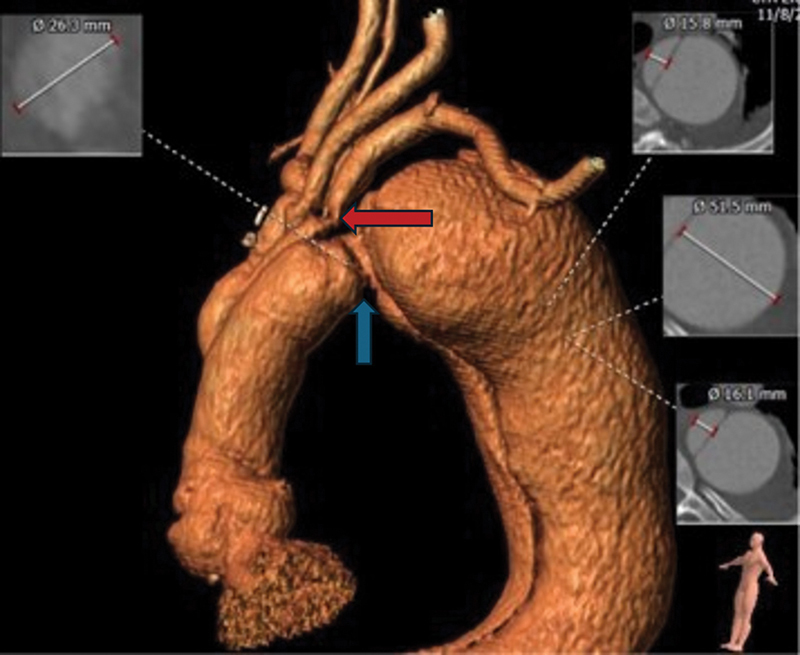
Computed tomographic angiogram after repair of Type A dissection and prior to repair of Type B aortic dissection. There is a stenosis at the graft to native aortic anastomosis (blue arrow) and a stenosis at the junction of the graft to left subclavian artery anastomosis (red arrow).

The patient is a 65-year-old male with a history of hypertension, tobacco abuse, and illicit drug use who presented with upper respiratory infection symptoms, chest and back pain, and was found to have a Stanford Type A, DeBakey Type I aortic dissection with 7-cm arch, and proximal descending aneurysm. Initial repair of the ascending aorta was delayed due to cardiomyopathy attributed to methamphetamine abuse, respiratory failure, pleural effusions, and acute kidney injury. Surgical repair was ultimately delayed for 10 months to allow recovery. The first stage of repair was grafting of the ascending aorta with elephant trunk into the descending aorta with separate grafts from the ascending graft to the innominate, left carotid, and left subclavian arteries ( Fig. 1 ). This procedure was done using cardiopulmonary bypass and hypothermic circulatory arrest. The patient's postoperative course after the first stage was complicated by pericardial effusion requiring a pericardial window and deep sternal infection requiring debridement and removal of sternal hardware.

**Fig. 2:**
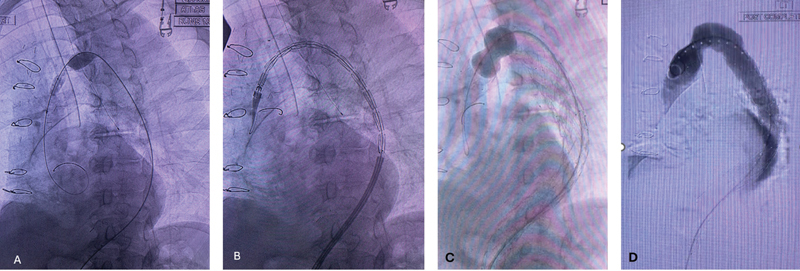
( **A** ) Dilation of prior ascending aortic graft to aorta anastomotic stenosis with 16-mm balloon. ( **B** ) Delivery of proximal thoracic endovascular aneurysm repair (TEVAR) device. ( **C** ) Balloon dilation of proximal TEVAR where it crosses the prior anastomotic stenosis. ( **D** ) Final proximal TEVAR angiogram.

Five months later, the patient underwent the second stage of treatment with placement of TEVAR stent grafts from the prior ascending graft to just above the celiac axis. This procedure was technically challenging due to the anatomy of the elephant trunk, narrowing of the brachiocephalic branch grafts as well as the graft-to-descending aortic anastomosis, and dissection into the iliac arteries, which made cannulation of the true lumen challenging. Ultimately, a brachiofemoral wire was needed, along with angioplasty of the graft-to-aorta anastomosis, to introduce the TEVAR devices ( Fig. 2 ). A lumbar drain was placed at the time of the procedure to reduce the risks of neurologic injury. This drain was eventually removed on the third postoperative day.

The patient was discharged home on the fifth postoperative day with near-normal renal function and no evidence of paraplegia or paraparesis. A computed tomographic angiogram (CTA) done 2 months after the second stage demonstrated excellent treatment of the ascending and descending components of the dissection without endoleak. Persistent filling of the false lumen was noted at the level of the distal thoracic aorta just above the visceral segment of the aorta at the distal extent of the TEVAR.

**Fig. 3:**
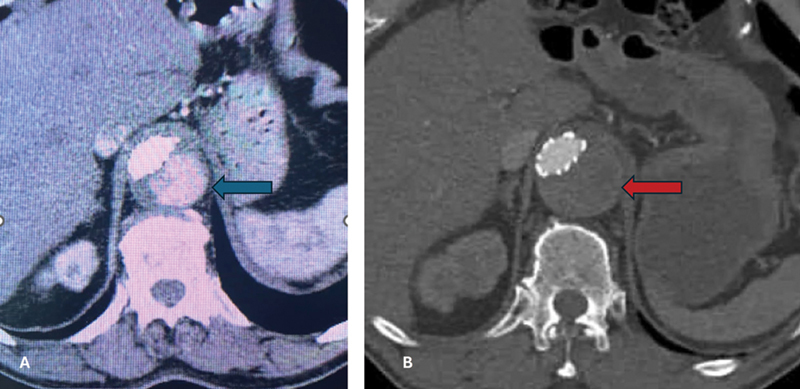
( **A** ) Postoperative computed tomographic angiogram (CTA) after thoracic endovascular aneurysm repair (TEVAR) shows persistent patency of distal false lumen (blue arrow) 2 months after procedure. ( **B** ) CTA at 20 months after repair at same level as in ( **A** ) showing interval thrombosis of false lumen (red arrow).

The patient subsequently presented 20 months after the second stage procedure with acute onset of paralysis. CTA done at the time of this presentation was largely unchanged compared with the one at 2 months postoperatively except for interval and subtle false lumen thrombosis noted in the distal thoracic aorta to the level of the renal arteries ( Fig. 3 ). Visceral, renal, and hypogastric arteries remained patent. Subsequent evaluation, including magnetic resonance imaging of the spine, concluded a complete loss of motor and sensory function in both legs at the T10 level due to spinal cord infarction.

## Discussion

SCI is a poorly understood and likely multifactorial complication of TEVAR. Hypotension, thrombosis, embolization, decreased spinal cord perfusion, and elevated cerebrospinal fluid (CSF) pressures are all patient-specific factors associated with increased risk for SCI. 10 11 12 13 From a surgical standpoint, prior aortic aneurysm repair, length of stent coverage, use of an iliofemoral conduit, and coverage of the left subclavian and hypogastric arteries are all factors implicated for increased risk of SCI. 11 12 13 14 The pathogenesis of delayed post-TEVAR SCI is also rather unique, given that patency of critical segmental arteries, covered by the stent graft, can be maintained not only by native collateral circulation, but also by residual flow through the false lumen. 15

Multimodal strategies involving early recognition and rescue intervention are emerging via SCI treatment algorithms. In 2021, the U.S. Aortic Research Consortium published guidelines recommending placement of a CSF diversion device with drainage permitted up to 30 mL/h, permissive hypertension to maintain mean arterial pressures (MAP) >90 mm Hg, and maintaining hemoglobin levels ≥10 mg/dL in order to reduce SCI occurrence. 16 Other adjunctive strategies include naloxone to reduce spinal metabolism, distal aortic perfusion, and intercostal artery reimplantation. 17 18 19 The ideal protocol for postoperative surveillance remains undefined; however, Tenorio et al 20 recently published a low rate of 1% permanent paraplegia among 170 patients undergoing fenestrated-branch endovascular aortic repair using a standardized preventative protocol of multimodal interventions and follow-up with computed tomography and duplex ultrasound of the renal-mesenteric arteries at 2, 6, 12 months and then annually thereafter during the first 5 postoperative years.

This case report contributes to a sparse body of literature, describing delayed post-TEVAR SCI at 20 months postoperatively. 10 Following complex two-stage reconstruction of his thoracic aorta, our patient's postoperative recovery period did not reveal any immediate concerns for neurologic deficits. His CSF drain was removed without issue on postoperative day 3 after he was determined to have adequate hemodynamics, with MAP > 90 mm Hg and Hgb >10 g/L. Follow-up CTA at 2 months postoperatively demonstrated a large amount of intraluminal thrombus within the dissected aorta without endo leak, consistent with the intended goals of therapy. He remained asymptomatic up to his 5-month telehealth appointment, with intention to repeat his imaging at 6 months.

While it is unclear what triggered his delayed SCI at 20 months postoperatively, it is possible that his remaining uncovered segmental branches had occluded following false lumen thrombosis. Kelly et al 10 previously described the longest time elapsed between TEVAR and onset-of-neurologic deficits at 19 months, with the authors also attributing their patient's delayed SCI to false lumen thrombosis. In contrast to our patient, his spinal cord insult was more diffuse and reversible and responded to intervention with spinal fluid drainage. The potential impact of postoperative imaging surveillance in this delayed presentation of neurologic insult is unknown, as the bulk of patients who develop interval false lumen thrombosis do not develop an acute neurologic event. This scenario also runs counterintuitive to conventional treatment goals, as false lumen thrombosis is generally considered a desired outcome of TEVAR. Nevertheless, it is a reminder for the vascular surgery community to maintain heightened vigilance for any potential contributing factors of delayed post-TEVAR SCI on a case-by-case basis.

## Conclusion

We describe an unusually delayed case of SCI at 20 months following TEVAR, with complete paralysis of bilateral lower extremities attributed to false lumen thrombosis. This experience highlights ongoing elusive challenges in the management of complex Type B aortic dissection (TBAD), as the implicated cause of this patient's delayed paralysis is contradictory to the standard goals of therapy. Although extremely uncommon, this case highlights a devastating late outcome of management of complex TBAD, one with limited options for successful treatment and reversal of the neurologic event.
